# Incidence of patients leaving against medical advice in government-subsidized hospitals: a descriptive retrospective study

**DOI:** 10.11604/pamj.2022.42.163.35161

**Published:** 2022-06-30

**Authors:** Eddieson Astodello Pasay-an, Romeo Patague Mostoles, Sandro Costanilla Villareal, Reynita Biong Saguban

**Affiliations:** 1 College of Nursing, University of Hail, Hail City, Kingdom of Saudi Arabia,; 2 AJA Campus, University of Hail, Hail City, Kingdom of Saudi Arabia

**Keywords:** Leaving against medical advice, retrospective study, government-subsidized hospitals, Saudi Arabia

## Abstract

**Introduction:**

this study aimed to determine the prevalence of leaving against medical advice (LAMA) in the local context and the associated predictors to help develop effective strategies to reduce its likelihood.

**Methods:**

this study employed a retrospective approach using medical records of the 16233 patients between 2016 and 2020 at various government-subsidized hospitals in the Hail region of Saudi Arabia.

**Results:**

the prevalence of LAMA was the highest in 2019 (91.9%) and 2017 (21.45%) among insured and non-insured patients, respectively. Furthermore, it was the highest among patients aged 20-25 years and the lowest among patients aged 46 years and above. The incidence of LAMA was the highest (15.48% for males and 29.53% for females) in 2016. In 2016-2019, the most common reason for LAMA was “wanted medication only,” while in 2020, the “fear of infection with COVID-19” was the main reason. High blood sugar was the most common diagnosis among the patients under consideration during the study period. Significant association was found between LAMA and patient's insurance status (t = 4.3123; p < 0.002); however, no association was found between LAMA and age (t = -0.8748; p > 0.658) and gender of patients (t = 1.9008; p > 0.302).

**Conclusion:**

strategies such as developing a suitable environment for patients and taking due care of their needs, providing individual consulting services, enhancing staff relations, and providing support to patients in need are vital. The likelihood of LAMA can be minimized by informing hospitalized patients and their relatives about the adverse effects of LAMA.

## Introduction

The phenomenon of patients leaving the hospital against medical advice (LAMA) can be described as when patients leave the hospital despite the treatment teams´ expressed recommendations [1]. Though it is the patient's right to refuse the treatment offered by the hospital, due consideration shall be made to the patient's condition. Leaving the hospital against medical advice is extremely risky with possible immediate and long-term consequences and can pose substantial liability issues. According to Glasgow *et al*. [2], patients who leave without medical advice are likely to be readmitted to the hospital or even succumb to their untreated or partly treated medical condition. Such distressing prospects have necessitated particular attention from medical personnel who wish to provide the finest possible care to their patients.

A number of studies have reported a range of reasons for patients leaving the hospital without the recommendation of the healthcare team. For example, Nasir and Babalola [3] and Stern *et al*. [4] found that patients leave the hospital for many reasons, including, inter alia, financial concerns, personal, family, staff conflicts, and most crucially, dissatisfaction with medical care. In most cases, Paul *et al*. [5] found out that its frequency and causes vary according to the ailment, geographical region, and type of healthcare system. In countries where healthcare services are not accessible at the point of delivery, the LAMA phenomenon varies greatly. For example, financial limitations can sometimes lead to patients dismissing themselves as soon as they experience improvement [6].

Several developing countries, such as Saudi Arabia, are known for the free medical services offered by various government entities. In Saudi Arabia, the private sector plays a pivotal role in the effective delivery of healthcare services, with rising engagement. However, due to the country's severe socioeconomic issues, privatization of healthcare is viewed to improve efficiency, quality, and public satisfaction in the delivery of healthcare while assuring that the government can meet its public commitments [7]. In this context, this view is likely to increase the incidence of LAMA. Conversely, LAMA in Saudi Arabia has been studied by El-Metwally and colleagues [8], who found that the prevalence of leaving against medical advice was 1%. According to Alayed [9], the viable and efficacious strategies for dealing with this problem include effective communication, flexible routines, policies, and procedures, negotiable management options, appropriate clinical treatment, and extensive documentation of LAMA.

Investigating patients leaving against medical recommendations is critical to developing suitable and efficacious treatments to reduce or eliminate the adverse consequences. Indeed, studies have examined the effects of LAMA on future rates of treatment refusal. However, the patient's reasons for leaving the hospital have not been adequately ascertained in various regions, which is crucial because the population in many of these areas is still at risk (e.g., succumbing to an ailment) [10]. Furthermore, Alayed [9] noted that the medical significance of patients departing against medical practitioner's advice is mainly unexplored. Therefore, in this study, the researchers aimed to determine the prevalence of LAMA in the local context and the chief associated predictors such that adequate strategies can be developed to reduce the likelihood of LAMA in the future.

## Methods

**Design and setting:** this study employed a retrospective approach using medical records of the 16233 patients admitted to government-subsidized hospitals in the Hail region, Saudi Arabia, between 2016 and 2020. All the government hospitals, namely King Khalid Hospital, King Salman specialist hospital, Hail General Hospital, and Convalescent hospital, were included in this investigation.

**Data collection:** after receiving approval from the Ministry of Health (MoH) Institutional Review Board, the researchers sought approval from the directors of the participating hospitals. Upon receiving due approval, another letter was sent to the records section authority to retrieve the medical records of the patients. The researchers had taken all cases of Leaving against medical advice from the year 2016 to the year 2020 from the participating hospitals regardless of their diagnosis, status, gender, and reasons for LAMA. Data gathering was conducted between August and December 2022.

**Ethical considerations:** the study protocol was granted by the Institutional Review Board of the Ministry of Health (IRB-2021-32) Hail, Saudi Arabia. The researchers ensured the confidentiality of the data. Any identifying characteristics of the patients were strictly concealed. Only pertinent information were gathered, such as date of confinement and date of discharge against medical advice, diagnosis, sex, and age. The researchers did not encounter any bias in identifying which medical records are included, as the aim is to include all cases of LAMA defined in this study.

**Data treatment/analysis:** the amassed data were treated with Statistical Package for the Social Sciences version 25. Descriptive statistics such as frequency and percentage were used for the date of confinement and date of discharge against medical advice, reasons, insurance status, diagnosis, sex, and age. In addition, regression analysis was used to note the predictors associated with LAMA.

## Results

**Figure 1:**
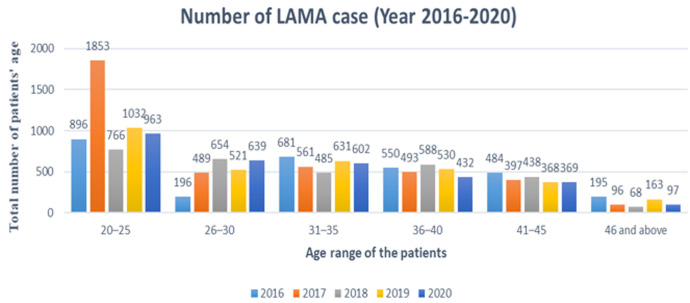
age of patients leaving against medical advice

**Figure 2:**
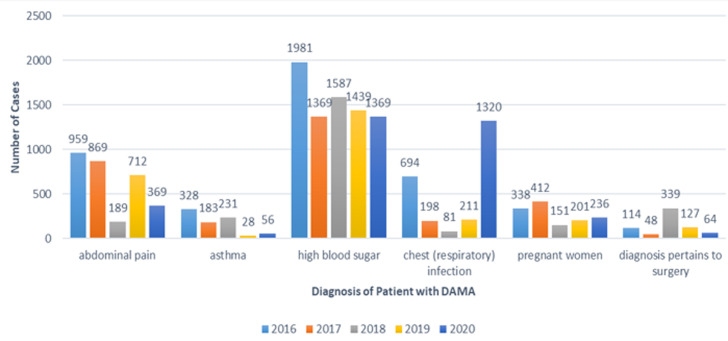
diagnoses of patients under consideration

Table 1 presents the percentage of patients who left against medical advice according to their insurance status. The prevalence of LAMA was the highest in 2019 (91.9%) among insured patients and highest in 2017 (21.45%) among non-insured patients. Figure 1 presents the trends in LAMA with regard to age in the study period. Evidently, during the study period, the prevalence of LAMA was the highest among patients aged 20-25 years old and the lowest among patients aged 46 years old and above. Table 2 shows the number of patients leaving against medical advice regarding their gender during the study period. The incidence of LAMA was the highest (15.48% for males and 29.53% for females) in 2016, while it was the lowest (7.19% for males and 13.90% for females) in 2018. The trend observed during the study period shows that most patients leaving against medical advice are male rather than female. Table 3 presents the reasons stated by various patients for LAMA. In 2016-2019, the most common reason reported for LAMA by the patients was “wanted medication only,” while in 2020, the “fear of infection with COVID-19” was the main reason. Figure 2 shows the different diagnoses among the patients under consideration. These diagnoses include abdominal pain, asthma, high blood sugar, respiratory infection, pregnant women, and surgery diagnosis. Overall, high blood sugar was the most common diagnosis throughout the study period. The logistic regression model suggests that, among the predictors, only insurance (t = 4.312; p< 0.002) was significantly associated with LAMA. The odds of being insured have increased by 3.8% (99% confidence interval (91.02-1.15)).

**Table 1:** percentage of patients leaving against medical advice according to their insurance status

Year	Frequency of LAMA incidents	Insured patients	Percentage (%)	Non-insured patients	Percentage (%)
2016	4,794	4,123	86.0	671	14.0
2017	3,645	2,864	78.6	781	21.4
2018	2,257	1,996	88.4	261	11.6
2019	3,241	2,978	91.9	263	8.1
2020	2,296	1,998	87.0	298	13.0

**Table 2:** gender of patients leaving against medical advice

Year	Total LAMA incidences	Male	Percentage (%)	Female	Percentage (%)
2016	4794	2,514	15.48	2,280	29.53
2017	3645	1,640	10.10	2,005	22.45
2018	2257	1,168	7.19	1,089	13.90
2019	3241	1,768	10.89	1,473	19.95
2020	2296	1,223	7.53	1,073	14.14
Total	16233	8313	51.19	7920	99.97

**Table 3:** reasons for LAMA stated by patients

Reasons for LAMA	2016	2017	2018	2019	2020
Wanted medication only	2,068	1,557	609	1,377	525
Cost of treatment for non-Saudi	785	403	531	712	691
Conflict between patient and physician	421	635	493	521	369
Patient went to specialized hospital	183	306	234	129	109
Fear of infection with COVID-19	0	0	0	0	1,381
Fear of the surgical procedure	144	48	27	127	30
Denial of the medical diagnosis	143	98	67	193	148
Wait time	300	425	123	166	152
**Total**	4,044	3,475	2,084	3,225	3,405

## Discussion

This study, conducted in the Hail region of Saudi Arabia, aimed to determine the prevalence of LAMA and its associated predictors to assist policymakers in developing interventions to reduce the likelihood of LAMA. This study showed that throughout the study period (2016-2020), the percentage of insured patients is growing, and indeed, many of the patients who opted for LAMA are insured. This implies that the insured patients were not provided with the highest quality care and facilities. In this context, it is necessary to monitor the supply of healthcare services to verify that services provided to insured customers meet the requisite standards. The findings agree with the study of Sharif *et al*. [11], Fenny *et al*. [12], and Dalinjong and Laar [13], affirming that most insured individuals had reported extended wait periods, verbal abuse, not being physically examined, and prejudice (e.g., racial). This warrants further investigation into why people are dissatisfied with service delivery. To promote confidence and satisfaction, the Ministry of Health must strive to provide regular monitoring and evaluation of provider services.

During the study period, the prevalence of LAMA was the highest among patients aged 20-25 years old and the lowest among patients aged 46 years old and above. The elderly's willingness to continue treatment may explain the low levels of LAMA in older age groups. In congruence with other studies [6,8,14,15], the age groups of 21-30 years have the highest rate of LAMA. In addition, review analysis of 61 papers reported that early age is a predictive factor for LAMA [1]. Practical measures such as providing middle-aged patients with counseling services, creating an appropriate environment for patients, and paying attention to their needs were effective.

The incidence of LAMA was the highest in 2016 and the lowest in 2018. However, most patients who left against medical advice are male rather than female. This finding agrees with other studies [8,14,16-18]. This observation essentially implies that due to their primary obligation of financial load, men have a larger sense of responsibility, a risk-taking attitude in making judgments, and a distinct advantage over women, who are more compliant with medical staff recommendations. In addition, in Saudi Arabia's patriarchal society, fathers are frequently the only custodians of their families' assets. This finding may explain why men feel a societal responsibility to care for their families. This implies a highly vulnerable patient population encountering significant non-compliance challenges to their health. Such findings can contribute to hospital strategies that should target male patients with any signs of LAMA during their stay to reduce their risk of non-compliance by speaking extensively about all aspects of care, minimizing conflict, and providing a kind and pleasant environment to the patient.

In this study, the reason for LAMA stated by patients was “wanting medication only,” which means that the decision to LAMA by a patient could indicate a breakdown in communication, which could be related to the number of patients, providers, and relationship problems. A few doctors, for instance, have remarked that by the time they are summoned to deal with the patient, the problem has already gone beyond their ability to have a positive impact. Participants in the nursing group, on the other hand, claimed that the extended period between a patient´s admission and a meeting with the attending physician frequently adds to a patient´s decision to LAMA. This finding conforms with those by Chan Carusone *et al*. and Hajek *et al*. [19,20], Beaulieu *et al*. [21], and Cheng *et al*. [22]. Such a result can contribute to the study of treatment refusal to learn more about the patient´s beliefs, expectations, anxieties, and personal requirements [20]. Starting with the initial interview, this procedure may demonstrate that patients do not lack knowledge but are just informed in different ways about their health [19]. If a patient still wants to leave prior to completing his/her treatment, patient liberty can be enhanced by facilitating the requisite post-hospital follow-up communication [23]. Beaulieu *et al*. [21] proposed a coordinated intervention effort, recognizing the joint role of patients and providers in the number of activities leading up to LAMA and actively seeking both patient and provider insight in developing the intervention, specifying action, and identifying chief performance indicators. A coordinated approach also builds on the issues and experiences from patient experiences about both natural and prevented LAMA, identifies and tackles provider group differences about who is most accountable for effecting change, and capitalizes on patients´ and providers´ mutual desire for improved outcomes [21].

In 2020, the most common reason for LAMA was the fear of COVID-19. This implies that patients who are afraid of contamination discontinue their therapy halfway through and leave without the recommendation of medical practitioners. This shows that the pandemic has impacted patients not infected with COVID-19, which should not be overlooked. According to Aydin and Doğan [24] and Demir *et al*. [25], it is reasonable to presume that sick people face an “avoidance-avoidance conflict” as a result of the anxiety caused by the danger of COVID-19 transmission when they are hospitalized. Patients who find themselves in a struggle between two undesirable situations may decide to terminate their treatment. It is suggested that healthcare professionals identify the dirty-clean zones of emergency services with sharp borders, and notifying patients may help reduce LAMA caused by fear of contracting COVID-19. Medical personnel should also keep in mind that people's religious beliefs in their work areas may influence their decision to undergo treatment.

It is worth noting that the most common diagnosis among the patients under consideration was high blood sugar. The result means that the urgency of care among patients with high blood sugar should be immediate. This finding is congruent with the study of El-Metwally *et al*. [8] and Edo *et al*. [26], showing that emergency cases are typically admitted to urgent care and are urged to stay in the hospital longer owing to their critical condition. Extended stays at the urgent care center would increase the patients' total medical expenditure, which may be challenging for them to bear. As a result, patients would choose LAMA in such instances, which could be one of the critical reasons for LAMA. It is recommended that hospitals raise public knowledge of diabetic patients and encourage them to seek timely treatment. In 2020, the rising of respiratory infection was noted to be the next most common diagnosis. The increasing case of respiratory infection could be related to COVID-19. This observation suggests that patients with respiratory infections were discharged from hospitals because they were concerned about contracting COVID-19. It is possible that they were handled by the same people or had the same specialty. According to Demir *et al*. [25], this could be because people with comorbidities believe they require greater medical attention. The more significant percentage of patients with a higher burden of respiratory infection in LAMA cases could be because these patients choose not to be in the hospital, where the risk of COVID-19 transmission is considerable [24]. This result may help inform the essential safeguards for all patients, particularly very ill patients, to feel safe at the hospital and receive proper treatment. The negative repercussions that may arise should be avoided by addressing the patients' and their relatives' concerns.

The insurance status was found to have a significant association with LAMA, which underlines that specific healthcare consumers, particularly those insured, are dissatisfied with the services provided. As remarked previously, several studies support this conclusion. Meanwhile, no association was found between LAMA and the age and gender of patients, which means that regardless of the age and gender of the patients, there is no change in their propensity for LAMA. Demir *et al*. [24] reported that age and gender were not influencing factors in the decision to leave the hospital. However, the finding opposes several studies by other scholars [8,14,16,27,28].

This study has a few limitations. For example, the data were gathered from government hospitals only, and private hospitals were excluded, which limited the generalization of the result in the region. Also, this study did not explore the area or department of LAMA in the hospital.

## Conclusion

The findings of this study can inform the policymakers on strategies such as developing a suitable environment for patients and taking due care of patients' needs, providing individual consulting services, enhancing staff relations, and providing support to patients in need are of vital importance. The likelihood of LAMA can be minimized by informing hospitalized patients and their relatives about the adverse effects of LAMA. The government should implement health insurance policies. Continued health education and an improvement in the population's socioeconomic situation may help minimize LAMA's prevalence and increase patients' chances of completing the treatment.

**Funding:** this research has been funded by the Scientific Research Deanship of the University of Ha’il, Saudi Arabia, through project number RG-21019.

### What is known about this topic

- Though it is the patient's right to refuse the treatment offered by the hospital, due consideration shall be made to the patient's condition;
- Patients who leave without medical advice are likely to be readmitted to the hospital or even succumb to their untreated or partly treated medical condition.

### What this study adds

- The prevalence of LAMA was the highest among patients aged 20-25 years old;
- Patients with insurance opted LAMA more than those who were not insured.
